# Probabilistic Linkage of Two National Databases to Study Emergency Care and Hospital Readmission in Children who Survive Traumatic Injury

**DOI:** 10.23889/ijpds.v1i1.352

**Published:** 2017-04-19

**Authors:** Tellen D Bennett, Peter E Dewitt, Elizabeth Juarez-Colunga, Lawrence J Cook

**Affiliations:** 1 Pediatric Critical Care, University of Colorado School of Medicine; 2 Biostatistics and Bioinformatics, Colorado School of Public Health

## Objectives

Trauma and, in particular, traumatic brain injury (TBI) cause enormous morbidity and mortality worldwide. Children who survive trauma have many health burdens, but how often those burdens lead to emergency care or hospital readmission is unknown. Readmissions occur frequently in adult TBI survivors: approximately 21-28% within one year. No single existing U.S. database contains the necessary variables to study readmissions of children hospitalized after trauma. We previously demonstrated that probabilistic linkage with Markov chain Monte Carlo parameter refinement could accurately link records of children with severe TBI without using protected health information. That work was presented at the IHDLN Conference in 2014. The objectives of this study are to 1) expand the previous linkage to include children with any type of trauma and 2) to determine the 1-year rates of Emergency Department (ED) visit and hospital readmission in children with TBI versus other trauma.

## Approach

Using the algorithm we reported previously, we linked the records of children hospitalized after trauma during 2007-2012 in the U.S. National Trauma Data Bank (NTDB) with the Pediatric Health Information System (PHIS) database.

The NTDB contains the Glasgow Coma Scale (GCS), necessary to categorize TBI. The PHIS database contains a unique identifier for each patient at each hospital, making longitudinal studies possible. Probabilistic linkage was performed using LinkSolv (Strategic Matching, Inc., Morrisonville, NY). We identified ED visits and hospital readmissions of the index cohort in the PHIS database through March 31, 2015.

## Results

Among children hospitalized after acute trauma for >= 2 days, we found that 83% of NTDB records linked accurately. Of the children hospitalized at 28 U.S. hospitals in 19 states, 49,477 were discharged alive; 15,438 had TBI. This represents approximately 10% of U.S. pediatric TBI hospitalizations during that period. The median age was 8 years and 64% were male. The 1-year hospital readmission rate was higher (12.2%, 283/2,306) in those with both TBI and other injuries than those with isolated TBI (8.5%, 1,120/13,132) or non-TBI injuries (8.1%, 2,790/34,039, p < 0.01 across groups). The 1-year ED visit rate differed only modestly between those three groups (14.1% versus 15.4% versus 14.2%, p < 0.01).

## Conclusion

This study leverages two linked national datasets to investigate emergency care and hospital readmission in children who survive trauma. Multivariable analyses are ongoing and will be completed by August 2016. We will generate hypotheses about potentially modifiable causes of emergency care and readmission.

